# Opening pathways of the DNA clamps proliferating cell nuclear antigen and Rad9-Rad1-Hus1

**DOI:** 10.1093/nar/gkt810

**Published:** 2013-09-12

**Authors:** Xiaojun Xu, Carlo Guardiani, Chunli Yan, Ivaylo Ivanov

**Affiliations:** Department of Chemistry, Center for Diagnostics and Therapeutics, Georgia State University, GA 30302, USA

## Abstract

Proliferating cell nuclear antigen and the checkpoint clamp Rad9-Rad1-Hus1 topologically encircle DNA and act as mobile platforms in the recruitment of proteins involved in DNA damage response and cell cycle regulation. To fulfill these vital cellular functions, both clamps need to be opened and loaded onto DNA by a clamp loader complex—a process, which involves disruption of the DNA clamp’s subunit interfaces. Herein, we compare the relative stabilities of the interfaces using the molecular mechanics Poisson−Boltzmann solvent accessible surface method. We identify the Rad9-Rad1 interface as the weakest and, therefore, most likely to open during clamp loading. We also delineate the dominant interface disruption pathways under external forces in multiple-trajectory steered molecular dynamics runs. We show that, similar to the case of protein folding, clamp opening may not proceed through a single interface breakdown mechanism. Instead, we identify an ensemble of opening pathways, some more prevalent than others, characterized by specific groups of contacts that differentially stabilize the regions of the interface and determine the spatial and temporal patterns of breakdown. In Rad9-Rad1-Hus1, the Rad9-Rad1 and Rad9-Hus1 interfaces share the same dominant unzipping pathway, whereas the Hus1-Rad1 interface is disrupted concertedly with no preferred directionality.

## INTRODUCTION

**Figure 1.**
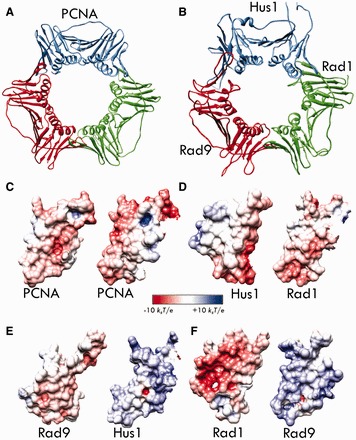
Common toroidal architecture of the DNA clamps PCNA and 9-1-1. (**A**) Structure of PCNA with the equivalent subunits shown in red, blue and green. (**B**) Structure of the checkpoint clamp with Rad9 shown in red, Rad1 in green and Hus1 in blue. (**C**) Surface electrostatics of the PCNA interface. (**D**) Surface electrostatics of the Hus1-Rad1 interface. (**E**) Surface electrostatics of the Rad9-Hus1 interface. (**F**) Surface electrostatics of the Rad9-Rad1 interface. Electrostatic potential is mapped onto the molecular surface for each interface and color-coded from red (negative) to blue (positive).

Genome duplication and maintenance are essential for all life, and the dynamic molecular machinery responsible for these vital biological functions is the replisome (1). Proliferating Cell Nuclear Antigen (PCNA) (2–4) and a related checkpoint protein, Rad9-Rad1-Hus1 (9-1-1) (5), are DNA clamps, which act as platforms for the assembly of core replisomal components on DNA (6). In this capacity, DNA clamps are essential in cellular activities ranging from DNA replication, repair of DNA damage, chromatin structure maintenance, chromosome segregation, cell-cycle progression and apoptosis (2,6–12). PCNA is a recognized master coordinator of multiple pathways controlling replication and DNA damage response. The clamp has a toroidal shape that wraps around DNA and topologically links the replicating DNA polymerase to its substrate. During lagging strand DNA synthesis, PCNA organizes three core replication proteins—DNA polymerase, Flap endonuclease 1 and DNA ligase I (13,14). Similarly, the checkpoint clamp 9-1-1 is a crucial constituent of complexes responsible for checkpoint signaling and base excision repair (BER). Although PCNA features three identical subunits, 9-1-1 (15–17) is heterotrimeric (Figure 1) reflecting the distinct roles of PCNA and 9-1-1 in DNA processing (18). Unlike PCNA, 9-1-1 does not associate with replicative polymerases but recruits checkpoint effector kinases to sites of DNA damage. 9-1-1 also stabilizes stalled replication forks (9,19,20) and stimulates BER enzymes (including Flap endonuclease 1 and DNA ligase I), thus linking BER activities to checkpoint coordination (21–24).

PCNA and 9-1-1 are composed of three subunits forming closed rings encircling DNA. Each subunit includes two wedge-shaped domains connected by a long inter-domain connector loop (IDCL) (3,4). Most partner proteins associate with the clamps through the IDCL using a consensus sequence called PIP-Box. The clamp subunits delimit an inner cavity whose walls are rich in positively charged residues needed for contacting DNA. The fact that PCNA and 9-1-1 form stable trimers implies an activated mechanism for loading onto DNA (25–28). A clamp-loader (AAA+ ATPase) opens and subsequently re-closes the clamps around DNA (29). Specifically, PCNA is loaded onto primer-template DNA by the pentameric replication factor C (RFC) complex (28–33). 9-1-1 is loaded by a variant clamp loader wherein the large Rfc1 subunit has been replaced by Rad17 (8,34) The clamp-loading mechanism is conserved in the three domains of life and involves opening of the pre-formed rings along a single subunit interface. As all three 9-1-1 interfaces are distinct, naturally, a question arises whether one interface is preferentially opened during clamp loading. Furthermore, as the protein machinery responsible for clamp loading is essentially the same for PCNA and 9-1-1 (besides the replacement of Rfc1 by Rad17), this prompts two important questions: (i) does subunit interface stability play a role in determining which interface is opened and (ii) is there an intrinsic difference in the energy required to open and load PCNA versus 9-1-1?

For the heterotrimeric 9-1-1 clamp, the question of which interface opens to allow a pre-formed ring to be loaded onto DNA junctions is still unresolved. There have been two competing proposals. First, Dore *et al*. argued that the Rad9–Rad1 interface serves as an opening gate during clamp loading. In their 9-1-1 structure (15) (PDB id: 3G65), the Rad9–Rad1 interface was found to be most polar and had the smallest buried surface area (BSA) (1100 Å^2^). By contrast, Sohn and Cho (16) independently solved the 9-1-1 structure (PDB id: 3A1J) and argued that the Hus1–Rad1 interface was most likely to open due to its close structural resemblance to the human PCNA interface. Finally, Xu *et al.* (17) published a third 9-1-1 crystal structure (PDB id: 3GGR) and proposed association between Rad1 and human Rad17 in the complex of 9-1-1 with the clamp loader. Owing to the chiral arrangement of the 9-1-1 subunits (Rad9, Rad1 and Hus1 in anticlockwise order viewed from the top), the Rad9 subunit would then be located beneath the Rfc5 subunit of the clamp loader. This would position the Rad9-Rad1 interface at the gap between Rfc5 and Rad17. Such positioning also implied the Rad9-Rad1 interface could be the gateway to open the checkpoint clamp.

Despite the availability of three independently determined 9-1-1 structures, this conundrum cannot be easily resolved for several reasons: (i) BSA is an imperfect indicator of interface stability; (ii) specific residue contacts at the interfaces differ among the three available structures; (iii) there are unresolved residues close to the interfaces in two of the three experimental structures (15–17). In this contribution, we analyze the DNA clamp interfaces to determine the interface serving as the opening gateway in 9-1-1. Specifically, we apply the MM/PBSA (MM/GBSA) method (35,36) in the AMBER package (37,38) to compute binding energies for the subunit interfaces in PCNA and 9-1-1, providing an unbiased comparison of relative interface stabilities. Furthermore, pairwise decomposition of the MM/GBSA binding energies allowed us to delineate the significant interactions contributing to the stability of each interface. Finally, by analyzing series of multiple-trajectory steered molecular dynamics (SMD) runs (pulling runs), we characterized the mechanical properties of the DNA clamp interfaces and their breakdown mechanisms under external forces.

### MATERIALS AND METHODS

We adopted a three-step computational strategy to evaluate the clamp interfaces. First, the molecular mechanics Poisson − Boltzmann (Generalized Born) solvent accessible surface area (MM/PBSA or MM/GBSA) method was applied to calculate binding energies and compare the relative stabilities of the four subunit interfaces of human PCNA and 9-1-1. Second, the computed binding energies were decomposed to reveal aggregate per residue contributions to interface stabilization. We also carried out pairwise MM/GBSA decomposition to construct a matrix of pair interaction energies for residues forming each interface. Third, we performed multiple-trajectory SMD simulations to monitor the pattern of breakdown for all energetically significant residue contacts identified in the MM/GBSA pairwise decomposition. Description for each of the three stages in our modeling protocol is given later in the text.

### Systems setup and molecular dynamics

The crystal structures of PCNA (4) (PDB ID: 1VYM) and Rad9-Rad1-Hus1 (15–17) (PDB ID: 3A1J, 3GGR, 3G65) were obtained from the Protein Data Bank. Molecular dynamics simulations before MM/PBSA analysis were performed using the intact trimeric clamps. Unresolved residues in the 3G65 and 3A1J structures were fixed based on the 3GGR structure. For the subsequent MM-PBSA calculations, we removed one clamp subunit to ensure only one interface (between the remaining two subunits) was evaluated. To limit computational expense, we carried out all SMD runs on reduced models, wherein we retained only the N-terminal domain of the first subunit and the C-terminal domain of the neighboring subunit. This approach is justified by the outcome of the MM/GBSA analysis, which showed no substantial contributions to the binding energies from residues outside the two adjacent domains defining each interface. The XLeap module of AMBER 9 (37,38) was used to add hydrogen atoms. All ionizable side chains were assigned to their ionization states at pH 7.0 using the WHATIF server (43). Each system was solvated with TIP3P water molecules (44) leaving a minimum distance of 10.0 Å from the protein surface to the edge of the simulation box. Counter ions were added to achieve charge neutralization, and additional 100 mM NaCl concentration was introduced to mimic physiological conditions.

The systems were minimized for 5000 steps with fixed backbone atoms followed by 5000 steps of minimization with harmonic restraints on the protein backbone atoms (k = 25 kcal/mol) to remove unfavorable contacts. All systems were then gradually heated to 300 K over 200 ps in the NVT ensemble while keeping the protein backbone constrained. The equilibration was continued for another 1.0 ns in the NPT ensemble, and the harmonic restraints were gradually released.

Production runs were carried out in the isothermal isobaric ensemble (1 atm and 300 K) for 20 ns for the intact clamp systems (for MM/PBSA). Long-range electrostatic interactions were evaluated with the smooth particle mesh Ewald algorithm (45). For the short-range non-bonded interactions, we used a cutoff of 10 Å with a switching function at 8.5 Å. The integration time step was 2 fs, and the bonds between hydrogen and heavy atoms were fixed to eliminate the most rapid oscillatory motions. The r-RESPA multiple time step method (46) was adopted with a 2 fs time step for bonded, 2 fs for short-range non-bonded interactions and 4 fs for long-range electrostatic interactions. All simulations were performed using the NAMD 2.7 code (47,48) with the AMBER Parm99SB parameter set (49) containing the force field for nucleic acids and proteins. Data were analyzed using the PTRAJ utility in AMBER (38) and custom VMD TCL scripts (50).

### Binding energy calculation

To calculate binding energies for all four clamp interfaces, we used the molecular mechanics Poisson − Boltzmann solvent accessible surface area (MM/PBSA) method (35,36). For the purposes of MM/PBSA analysis, we sampled frames at 4 ps intervals from the last 10 ns of the MD trajectories (the first 10 ns were discarded as equilibration). In total, 2500 frames were used for averaging. Free energy of binding can be computed as follows:

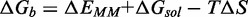

where ΔE_MM_ represents gas-phase molecular mechanics binding energy (van der Waals and electrostatics); ΔG_sol_ is the change in solvation free energy, and ΔS is the gas-phase entropy change on binding. The electrostatic solvation energy contribution to ΔG_sol_ can be estimated using the finite difference Poisson − Boltzmann (PB) method. A grid size of 0.5 Å was used in the PB calculation, and the dielectric constants for protein and water were set to 1.0 and 78.0, respectively. The non-polar contribution to ΔG_sol_ was estimated by the solvent-accessible surface area according to equation:

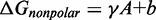



The solvation parameters γ and b are set to be 0.0072 kcal mol^−1 ^Å^−2^ and 0 kcal mol^−1^, respectively. The surface area A was calculated using MolSurf in AMBER 9 (38). The probe radius of the solvent was set to 1.4 Å. The optimized set of atomic radii in AMBER 9 was used, and the atomic charges of the protein were taken from the ff99SB force field. The entropy contribution was not included, and, therefore, the computed values for the binding energies should be considered only relative to one another. Computed relative binding energies represent an appropriate measure of interface stability under the assumption that the gas-phase entropy terms are similar for the PCNA and 9-1-1 interfaces. The similar extent and secondary structure of the four clamp interfaces justifies such an approach.

Finally, we carried out two types of binding energy decomposition calculations. First, aggregate contributions of individual residues toward interface stability were evaluated (1D decomposition). Second, a pairwise (2D) decomposition was carried out to pinpoint residue pairs contributing significantly (above −1.0 kcal/mol for PCNA and Hus1-Rad1; −2.0 kcal/mol for Rad9-Hus1 and Rad9-Rad1) toward the interface binding energy. To reduce the computational expense of the pairwise decomposition, we used model truncation analogous to the one used in SMD. The MM/GBSA decomposition follows an established method to estimate the contributions of residues from each of the two subunits to the total binding energy by means of component analysis (41). PTRAJ module of AMBER TOOLS 12 and VMD (50) were used for the analysis of trajectories and structural visualization.

### SMD

To examine the mechanical properties of the subunit interfaces in PCNA and 9-1-1 and establish interface disruption mechanisms under external forces we relied on the SMD method (51). To initiate the SMD runs, we selected snapshots from preliminary 10 ns unbiased simulations of PCNA and 9-1-1 (3A1J) at equal intervals along the trajectories. These statistically uncorrelated configurations were used to initiate constant velocity SMD. In each interface, we have applied constraints to the β-strand of the N-terminal domain while applying a harmonic force *k* = 15 kcal mol^−^^1 ^Å^−^^2^ to the center of mass of the other strand. The pulling direction was along the line connecting the centers of mass of the two domains. The constant pulling velocity was 1 Å ns^−^^1^, and the pulling run had a total duration of 12 ns so as to achieve an opening of 12 Å of the interface. During the pulling trajectories, 1200 structures were sampled at intervals of 10 ps for analysis. In total, the SMD simulation time exceeded 1.4 µs necessitating the use of extensive supercomputing resources. Thus, the project required >1 million CPU hours at two supercomputing facilities (NICS Kraken and NERSC Hopper II supercomputers).

Analysis of the SMD runs was carried out by following the time evolution of the contacts identified as significant contributors to binding in the pairwise (2D) MM/GBSA decomposition (41). The contacts were classified as hydrogen bonds, salt-bridges, hydrophobic interactions and suitable cutoffs applied to determine the presence of the contact in the trajectory frames. For each contact detected during the pulling trajectory, the time of last occurrence (allowing for the possibility of contact breaking and reforming) was used to identify the breakdown time. The interface opening mechanism could then be followed on a contact map wherein the color-coding scheme signified groups of contacts cleaved within the same time interval.

## RESULTS AND DISCUSSION

### Rad9-Rad1 interface requires least amount of energy to open

To understand the structural and energetic basis for interface stability in PCNA and 9-1-1, we analyzed all four subunit interfaces (PCNA-PCNA, Rad9-Rad1, Rad9-Hus1 and Hus1-Rad1) through MD simulations along with the application of the MM/PBSA method (35,36). We use complete and fully relaxed models of the trimeric sliding clamps (PDB id: 3GGR, 3A1J and 3G65) (15–17) embedded in an aqueous solvent environment. Subunit interface binding energies were averaged over 2500 independent conformations. Various components of the subunit interaction energy ΔG_b_ were evaluated for all three available 9-1-1 X-ray structures (Table 1, Supplementary Tables S1 and S2). When MM/PBSA is applied to extended interfaces, it is challenging to accurately assess the gas-phase entropic contribution. Therefore, quantitative comparison of the interfaces involved only the enthalpic contribution, and the values in Table 1 and Supplementary Tables S1 and S2 represent binding energies rather than free energies. As expected, due to lack of entropy-enthalpy compensation, the absolute binding energies are overestimated. Nonetheless, previous applications of MM/PBSA to protein interfaces have shown the usefulness of comparing the relative stabilities of binding epitopes and identifying binding hotspots based on energy decomposition (39,40).

**Table 1.** Binding energy analysis (kcal mol^−1^) for the DNA clamp interfaces in PCNA and 9-1-1 (3GGR model)

Contribution	3GGR			
	PCNA/PCNA	Rad9/Hus1	Hus1/Rad1	Rad9/Rad1
Δ*E*_ele_	866.04 (46.68)	−479.45 (88.28)	−38.87 (37.04)	−584.25 (53.97)
Δ*E*_vdw_	−80.65 (5.38)	−98.14 (7.77)	−92.49 (6.27)	−80.12 (6.12)
Δ*G*_non-polar_	−11.68 (0.42)	−16.58 (0.70)	−12.62 (0.55)	−12.84 (0.46)
Δ*G*_polar_	−827.27 (46.05)	502.33 (84.81)	69.78 (34.50)	618.16 (50.82)
Δ*G*_sol_^a^	−838.95 (45.83)	485.75 (84.34)	57.17 (34.42)	605.31 (50.74)
Δ*G*_ele_^b^	38.77 (10.49)	22.88 (10.65)	30.91 (7.73)	33.90 (10.94)
ΔG_b_	−**53.56** (8.91)	−**91.84** (9.08)	−**74.19** (8.16)	−**59.06** (8.68)
ΔG_b_ Ratio	**1.00**	**1.71**	**1.39**	**1.10**
BSA (Å^2^)	1555	2117	1629	1645

^a^Polar/non-polar (Δ*G*_sol_ = Δ*G*_polar_ + Δ*G*_non-polar_) contributions to Δ*G*_b_.

^b^Electrostatic (Δ*G*_ele_ = Δ*E*_ele_ + Δ*G*_polar_) contributions to Δ*G*_b_. Calculation of Δ*G*_b_ does not explicitly consider entropy contributions. Standard deviations are shown in parentheses. Averaged BSA for the interfaces are given units of Å^2^.

From the data in Table 1 (also Supplementary Tables S1 and S2), we immediately conclude that the 9-1-1 interfaces differ substantially in stability. For the most complete structure (3GGR), the computed binding energies ΔG_b_ establish the following order of stability: Rad9-Hus1 > Hus1-Rad1 > Rad9-Rad1 ≈ PCNA-PCNA (ratios of ΔG_b_ of 1.71:1.39:1.10:1.0). This ordering is maintained regardless of which X-ray structure was used in constructing the model (e.g. for 3G65 the ΔG_b_ ratios are 1.86:1.49:1.16:1.0). The only outlier value is the Rad9-Hus1 binding energy in the 3A1J model. Missing residues at this interface were added by homology modeling (using 3GGR as template) likely leading to a marginally lower ΔG_b_. Comparison among the independently set-up 3G65, 3A1J and 3GGR models reveal that the binding energy ratio ΔG_b_ (Rad9-Rad1)/ΔG_b_ (PCNA) is remarkably consistent, irrespective of the initial structural model (ratio of 1.16, 1.12 and 1.10, respectively). The Rad9-Rad1 interface is the weakest and, thus, a clear choice to be the gateway for opening the checkpoint clamp. Furthermore, the binding energy of Rad9-Rad1 is practically indistinguishable from the binding energy of the PCNA-PCNA interface. Therefore, the energetic requirement for the Rad17-RFC2–5 complex to open 9-1-1 is essentially same as the requirement for RFC to open the PCNA ring. From an evolutionary standpoint, this outcome implies 9-1-1 and PCNA may have been subject to evolutionary pressure to optimize the overall stability of one subunit interface (Rad9-Rad1 or PCNA-PCNA) to match the requirements of the clamp loading machinery.

Despite the almost perfect match in overall binding energies, the stability of Rad9-Rad1 and PCNA-PCNA interfaces is achieved by different means. The PCNA interface is largely flat and hydrophobic and does not display electrostatic complementarity between the opposing binding epitopes (Figure 1C). By contrast, the Rad9-Rad1 interface features more polar contacts and a shorter hydrophobic patch between the two antiparallel β-strands S9-S13 (16) (Figure 3B). The binding surfaces from the Rad9 and Rad1 subunits display high electrostatic complementarity (Figure 1F) as reflected in the computed gas-phase electrostatic energy. ΔE_ele_ is favorable for the Rad9-Rad1 interface but unfavorable for the PCNA interface (Table 1). By contrast, the electrostatic solvation energy (ΔG_polar_) disfavors Rad9-Rad1 binding, whereas it favors PCNA interface formation. Rad9-Hus1 and Hus1-Rad1 also display favorable ΔE_ele_ due to electrostatic complementarity between the 9-1-1 subunits (Figure 1D–F)—a feature, which is absent in the PCNA interface.

Finally, it is notable that the computed BSA values for the interfaces averaged over the simulation trajectories (Table 1, Supplementary Tables S1 and S2) do not correlate well with the computed binding energies. Using only ΔG_non__-__polar_ (or alternatively BSA) to evaluate the 9-1-1 interfaces would suggest Hus1-Rad1 to be marginally less stable than Rad9-Rad1, inverting the order of stability established through the total binding energies ΔG_b_. However, interface formation is driven by both polar (ΔG_polar_ and ΔE_ele_) and non-polar interactions (ΔG_non__-__polar_ and ΔE_vdw_), which in the balance determine ΔG_b_. As BSA accounts only for the ΔG_non__-__polar_ contribution, it is a poor gauge of relative interface stability.

### Structural determinants of PCNA and 9-1-1 interface stability

**Figure 2.**
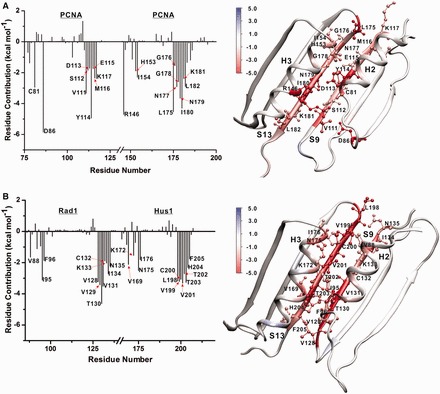
Origins of interface stability for the PCNA and Hus1-Rad1 interfaces from 1-D MM/GBSA decomposition analysis. (**A**) Aggregate binding energies for the individual residues of the PCNA interface (left) and residue contributions mapped onto the structure (right). (**B**) Aggregate binding energies for the individual residues of the Hus1-Rad1 interface (left) and residue contributions mapped onto the structure (right). Only residues contributing above a ±1.5 kcal mol^−1^ threshold in ΔG_b_ are represented.

**Figure 3.**
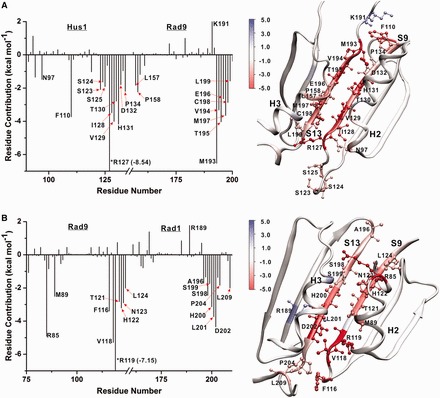
Origins of interface stability for the Rad9-Hus1 and Rad9-Rad1 interfaces from 1-D MM/GBSA decomposition analysis. (**A**) Aggregate binding energies for the individual residues of the Rad9-Hus1 interface (left) and residue contributions mapped onto the structure (right). (**B**) Aggregate binding energies for the individual residues of the Rad9-Rad1 interface (left) and residue contributions mapped onto the structure (right). Only residues contributing above a ±1.5 kcal mol^−1^ threshold in ΔG_b_ are represented.

To delineate the structural features that affect 9-1-1 interface stabilities, we decomposed the total binding energies ΔG_b_ into aggregate per residue contributions (1D decomposition) (41). All residues contributing substantially toward interface stabilization/destabilization above a ±1.5 kcal mol^−^^1^ threshold are shown in Figures 2 and 3. Residue contributions are also color mapped onto the structure of the respective interfaces. First, we note that all interfaces in 9-1-1 and PCNA are stabilized entirely by local interactions with no above-threshold contributions arising from residues outside the adjacent N-terminal and C-terminal domains. Despite dissimilar sequences, the 9-1-1 and PCNA interfaces share common structural features (4,15). At the core of each clamp interface is an antiparallel β-sheet (strands S9 and S13), forming part of the outer shell of the trimeric PCNA or 9-1-1 ring. This β-sheet is reinforced to varying degrees by two adjacent α-helices (labeled H2 and H3) facing toward the central cavity of each clamp. Such an arrangement of secondary structure elements imparts structural stability to the clamps. Residue contacts in the loop regions above and below the central β-sheet contribute with varying extent to the overall binding energy of each interface. The planar ring architecture results in the clamps being two-sided: the front (top) face exposes the IDC loops and is thus responsible for binding most replication factors (notably DNA polymerases and the clamp loader). The back (bottom) side of PCNA is characterized by three loops denoted as P-loops (or guide loops). The 9-1-1 interfaces likewise have a front and back side, and in the analyses that follow, we have oriented all interfaces so that the IDCL-presenting face is denoted as ‘top’ and the back face is ‘bottom’. Classification of the opening pathways follows the same convention.

In Figure 2, we first compare the interfaces PCNA-PCNA and Hus1-Rad1, which were suggested by Sohn and Cho (16) to be structurally most similar. Indeed, both interfaces are flat and hydrophobic, which is clearly reflected in the presence of two prominent largely symmetric peaks in the binding energy graphs. The peaks correspond to the two antiparallel β-strands at the core of the PCNA or Hus1-Rad1 interface and together account for most of the computed ΔG_b_. The roughly symmetric peaks suggest that there is no preferred directionality to open these two interface from either the top or the bottom side.

For PCNA the central β-sheet (V111-K117 and L175-L182) is the primary determinant of interface stability with 15 residues having above-threshold contributions. The interface is stabilized by the presence of seven stable backbone hydrogen bonds in a largely hydrophobic sequence context. The residues in the middle of the sheet contribute more significantly to ΔG_b_ than the flanking residues at the edge of the sheet. There are very few residues outside the β-sheet with above-threshold contributions. Interactions between the adjacent helices H2 and H3 are considerably less substantial, except for a single salt bridge between residues D86 and R146 at the back of the interface. Together, these two residues add −10.56 kcal/mol to the total binding energy. Three additional residues C81, H153 and I154 from helices H2 and H3 also contribute −2.95, −1.89 and −2.30 kcal/mol, respectively.

While the Hus1-Rad1 interface superficially resembles the PCNA interface (16 contributing residues in the core β-sheet; V128-N135 and L198-F205), the average computed binding energy per residue within the β-sheet is higher (−2.91 kcal/mol) for Hus1-Rad1 compared to PCNA-PCNA (−2.65 kcal/mol). Additionally, there are significantly more hydrophobic residues from the adjacent H2 and H3 helices contributing above the threshold level (V88, I95, F96, V169, N175, I176). Such differences in the extent of the hydrophobic contacts justify the observed order of stability with the PCNA-PCNA interface being easier to open than Hus1-Rad1.

In Figure 3, we compare the two more polar interfaces–Rad9-Hus1 and Rad9-Rad1. Once again, interactions within the central β-sheets play the most prominent role (R127-P134 and M193-L199 for Rad9-Hus1; V118-L124 and A196-P204 for Rad9-Rad1), resulting in two primary peaks in the 1-D MM/GBSA decomposition graphs. In contrast to PCNA-PCNA and Hus1-Rad1, the peaks in Figure 3 are largely asymmetric, featuring a broader distribution of binding energies. Stronger contacts are clustered at the bottom of the two interfaces. These stronger contacts are likely to break late during the interface rupture induced by the clamp loader, whereas weak contacts would break first. Therefore, the decomposition of ΔG_b_ (either 1D or pairwise 2D) gives us a clear prediction of the preferred direction for opening the subunit interfaces under external forces. The observed asymmetry of the peaks for Rad9-Rad1 and Rad9-Hus1 is indicative of preferred direction to open both interfaces from the top side. This trend is especially well pronounced for Rad9-Rad1, which is also the most polar of the three 9-1-1 interfaces. However, there are also substantial differences between Rad9-Rad1 and Rad9-Hus1 in terms of the number and type of residues contributing to ΔG_b_. Rad9-Rad1 is clearly the weaker interface due to fewer contacts within the core β-sheet and only two above-threshold contributions from outside the β-sheet: residues M89 and R85 from helix H2. By contrast, the Rad9-Hus1 interface features not only exceptionally strong contributions within the core β-sheet (per residue average of −3.51 kcal/mol) but also many contributing residues outside the core of the interface—N97 from helix H2, L157 and P158 from helix H3, F110 and S123-S125 from loop regions of the interface. Thus, Rad9-Hus1 appears to be the most substantial of all 9-1-1 subunit interfaces.

### Clamp opening pathways under external force

To address the mechanisms and pathways for clamp opening, we monitored subunit interface disruption under external forces. This was accomplished by multiple-trajectory SMD runs using truncated PCNA and 9-1-1 models. Truncation to include only the N-terminal and C-terminal domains from each interface is fully justified by the MM/GBSA results, which showed the clamp subunits to be held together entirely by local interactions. The external forces were applied to pull apart the β-strand at the core of each interface. Visual classification of the SMD trajectories/opening pathways (Table 2) was performed before detailed contact analysis. Our SMD simulations showed that similar to the case of protein folding, the clamp opening for both PCNA and 9-1-1 does not occur through a single uniform breakdown mechanism. Instead, the results are consistent with the existence of an ensemble of parallel clamp-opening pathways, some more prevalent than others. These pathways are characterized by specific groups of contacts that differentially stabilize the regions of the interface and, thus, determine the spatial and temporal patterns of breakdown. The stochastic nature of interface opening is consistent with recent work of Benkovic *et al.* (42), which pointed out that clamp loading proceeds by an inefficient and largely heuristic search. Instead of a single highly confined pathway, the process involves multiple attempts and many intermediates (possibly also off-pathway states). Thus, in addition to its primary function, the clamp loader has to recycle incorrectly loaded clamps.

**Table 2.** Classification of the DNA clamp opening pathways from SMD

Pathway classification	Subunit interface			
	PCNA/ PCNA	Rad9/ Hus1	Hus1/ Rad1	Rad9/Rad1
Top-down	12	16	9	17
Bottom-up	10	2	9	5
Concerted	8	4	1	0

Number of pathway classified as ‘top-down’, ‘bottom-up’ or ‘concerted’ for each clamp subunit interface are shown.

Despite the stochastic nature of clamp opening, the dominant pathways exhibit certain common features. Interface break-up almost invariably involves unzipping of the central β-sheet (strands S9–S13) followed by separation of the more loosely connected H2 and H3 helices and the loop regions of the interface. The unzipping of the β-sheet can occur in the top-down direction, bottom-up direction or by cooperative collapse of the contacts between the S9 and S13 strands. Therefore, in Table 2, we classify the opening pathways as ‘top-down’, ‘bottom-up’ or ‘concerted’. We note that the classification is based on the overall direction of opening for the entire interface (not just the β-sheet collapse). In a small number of SMD runs (<10%), the initial opening was followed by partial re-formation of the contacts at the interface. These runs were excluded from the pathway classification in Table 2.

**Figure 4.**
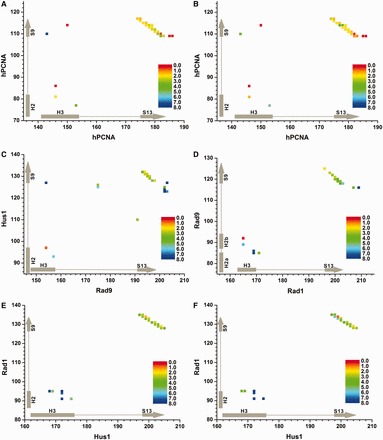
Dominant pathways for DNA clamp opening from contact analysis of SMD simulations. Time evolution of significant contacts for (**A**) the PCNA interface in the ‘top-down’ pathway; (**B**) the PCNA interface in the ‘bottom-up’ pathway; (**C**) the Rad9-Hus1 interface in the ‘top-down’ pathway; (**D**) the Rad9-Rad1 interface in the ‘top-down’ pathway; (**E**) the Hus1-Rad1 interface in the ‘top-down’ pathway; (**F**) the Hus1-Rad1 interface in the ‘bottom-up’ pathway. Averaged break-up times of above-threshold contacts from the pairwise MM/GBSA decomposition are color-mapped on the panels from red (0 ns) to blue (8 ns).

Under external forces the four clamp interfaces display distinct rupture mechanisms. For PCNA-PCNA and Hus1-Rad1 clamp opening in the ‘top-down’ or ‘bottom-up’ direction is equally probable. Indeed, for PCNA, all three pathways, including the concerted pathway, are equally represented. For Hus1-Rad1, there is a higher tendency for the opening process to start from the ends of the interface. However, once initiated, the unzipping is completed within a narrow time interval, which is indicative of almost cooperative disruption of the backbone hydrogen bonds at the interface core (Figure 4E and F). By contrast, the more polar Rad9-Rad1 and Rad9-Hus1 interfaces open preferentially in the ‘top-down’ direction. This outcome is fully consistent with our prediction from the 1D and pairwise MM/GBSA decompositions (Figures 2 and 3 and Supplementary Figure S1). The origin of preferred directionality lies in the uneven distribution of strong polar or charged contacts along these interfaces. Moreover, polar contacts found outside the central β-sheet exert more significant bias on the opening direction. The reason for this outcome is that these contacts involve specific side-chain interactions, whereas contacts within the core β-sheet occur mostly through the backbone and are thus non-specific. Preferred directionality is lost when the core of the interface is primarily hydrophobic and held together by backbone hydrogen bonds (as is the case for PCNA-PCNA and Hus1-Rad1).

Next, we carried out detailed analysis of the interface rupture mechanisms in PCNA and 9-1-1. To this end, we subdivided all pulling runs by type of opening pathway (Table 2) and analyzed the pattern of contact disruption as the interfaces were pulled apart. Only residue contacts contributing above-threshold in the pairwise MM/GBSA decomposition were considered and their time evolution monitored throughout the pulling trajectory. To follow the contact dynamics, we first classified the MM/GBSA residue pairs by type of interaction. For hydrogen bond contacts (backbone, side-chain H-bonds and direct salt bridges), we used heavy atom distance cutoff of 3.3 Å and an angle cutoff of 50°. Hydrophobic contacts were identified by considering whether heavy atoms from the first hydrophobic residue were in van der Waals contact with any heavy atom from the second hydrophobic residue. Polar residues not involved in hydrogen bonding were treated by applying the same distance criterion used for hydrophobic contacts. The time of last occurrence (within the specified cutoffs) of each MM/GBSA pair was recorded, and these times were averaged over the trajectories belonging to the same pathway. Naturally, identical contacts collapse at different times in different trajectories. However, we observed that within each pathway, the rupture events for specific contacts occurred within narrow time intervals, justifying the use of averaged break-up times in defining the opening mechanism.

Results from this analysis are shown in Figure 4 and reveal the characteristics of the dominant pathways for subunit interface disruption in PCNA and 9-1-1. Description of the contacts includes average break-up times in nanoseconds and interaction energies ΔG_b_ from the pairwise MM/GBSA decomposition (noted in parenthesis after each contact). For PCNA (Figure 4A and B), the opening mechanism is dominated by the disruption of the two antiparallel β-strands in the outer shell of the clamp. There is no clear distinction between the ‘top-down’ and ‘bottom-up’ pathways in terms of timing of contact disruption. Indeed, the majority of contacts between the β-strands disappears in a relatively narrow time interval from 1.5 ns to 4 ns, regardless of whether strand unzipping is initiated from the top or bottom side.

For the 9-1-1 interfaces, it generally took more time to initiate S9–S13 strand separation. This is illustrated by the shift in color toward the green/blue end of the scale as compared with PCNA in overall agreement with our ΔG_b_ results. Pulling the Hus1-Rad1 interface did not result in a single dominant pathway (Figure 4E and F) consistent with the flattened binding energy profile in the 1D or pairwise 2D MM/GBSA decompositions. Rupture of the core β-sheet in Hus1-Rad1 occurred with no obvious directionality and within an even narrower time range compared to PCNA: 2.9 ns—3.6 ns for the top-down pathway and 2.9 ns—4.0 ns for the bottom-up pathway. The pair Val199–Lys133 (−1.76 kcal/mol) lasted longer than any other β-sheet contact (5.8 ns) owing to the long flexible side chain of Lys133 reaching across the interface. The strongest contacts from the H2, H3 α-helices N175-D91 (−4.54 kcal/mol), K172-D91 (−4.05 kcal/mol) and K172-S94 (−2.80 kcal/mol) lasted beyond the complete separation of the β-strands in both pathways (rupture times of 8.2, 8.1 and 7.5 ns, respectively). Therefore, helix separation on the inner side of the interface occurred after the rupture of the core β-strands. We conclude that just like in PCNA, the Hus1-Rad1 interface opens by cooperative non-directional unzipping of the core β-strands dominated by disruption of hydrophobic contacts.

The top-down pathway was the dominant mechanism for opening the Rad9-Hus1 interface (Figure 4C). Disruption of the core β-strands for this interface occurred in a much broader time range (2.0–7.0 ns) compared with PCNA, indicating the presence of persistent contacts whose break-up requires much larger external forces. This notion is consistent with Rad9-Hus1 being the most stable of all 9-1-1 interfaces. The strongest interaction identified in the pairwise decomposition was between charged residues D203–R127 (−10.8 kcal/mol) and was positioned at the extreme back side of the interface. Not surprisingly, this was the last contact to break at 10.6 ns. Other robust contacts included E154-R127 (−4.12 kcal/mol), E202-S125 (−4.45 kcal/mol), E202-Ser124 (−5.62 kcal/mol), D203–S123 (−2.66 kcal/mol) and L157-K93 (−3.38 kcal/mol), which all resided in the bottom region of the interface and cooperatively resisted separation until average break-up times well above 6.5 ns. All of these pairs included polar or charged residues, corroborating the importance of charged interactions in maintaining interface stability and establishing a predominant opening direction.

The opening mechanism of the Rad9-Rad1 interface (Figure 4D) revealed an even more pronounced tendency for directional opening as compared with Rad9-Hus1. Rupture of the interface was initiated from the top side, which incidentally is also the side binding the clamp loader. Thus, it is attractive to speculate that the Rad9-Rad1 interface is weakest precisely at the point of application of mechanical force by the clamp loader. A group of resilient pairwise interactions at the bottom of the interface is responsible for the observed ‘top-down’ directionality of opening. These interactions feature both charged and hydrophobic residue contacts: R119-D202 (−16.50 kcal/mol), R85-E169 (−6.55 kcal/mol), F116-L209 (−2.54 kcal/mol), F116-S207 (−2.66 kcal/mol) and V118-Y203 (−2.98 kcal/mol). The strongest contact R119-D202 resides at the lower end of the β-sheet and along with V118-Y203 did not break until a very late stage (5.8 ns) of interface separation. Phe116-Leu209 was typically the last contact to rupture (11.7 ns) in most of the SMD trajectories. Despite the overall top-down rupture pattern, there were a couple of persistent contacts at the top of Rad9-Rad1. These involved the salt bridge R85-E169 (−6.55 kcal/mol) and the S86-E169 pair, both located at the top of the H2, H3 α-helices. Maintaining these two contacts until the latter stages of interface separation (7.8 ns) was made possible by the long side chains of Arg85 and E169, which could reach across the partially opened S9–S13 β-strands. Once again, the results highlight the importance of salt bridges in determining the interface opening mechanism and for the overall architecture of the DNA clamps.

### Concluding remarks

The toroidal architecture adopted by DNA clamps is a perfect example of how molecular shape follows function. Despite low sequence similarity, clamps from bacteriophage to archaea and humans all form trimeric rings encircling DNA. The function of these protein rings is to serve as mobile scaffolds in the coordinated assembly of the DNA replication and repair machinery. In this case, function requires a subtle balance between the ability to form stable trimers and the necessity to open to be loaded onto DNA. All clamps use a common loading mechanism, wherein a single subunit interface is ruptured by the clamp loader to allow threading of primer-template DNA and followed by closure of the ring.

Why have two distinct clamps, PCNA and 9-1-1, evolved in humans? One may speculate that the purpose of the heterotrimeric 9-1-1 is to ensure selective recruitment of different partners to different subunits on the same ring-shaped platform. This cannot be accomplished by PCNA, except in a stochastic manner because the PCNA subunits are equivalent. Importantly, the different subunits of 9-1-1 not only differentiate the clamp from PCNA but also open up the possibility that affinity differences among the clamp interfaces may have functional significance. Specifically, it has been proposed that the Rad9-Rad1 interface of the checkpoint clamp is the weakest and, therefore, serves as the opening gate for loading 9-1-1 onto chromatin. However, this proposal remains controversial with others positing Hus1–Rad1 is the opening gate based on structural resemblance to the PCNA interface. Through detailed subunit interface analysis, we identified Rad9-Rad1 as having the lowest affinity among all 9-1-1 interfaces. This finding makes Rad9-Rad1 a clear candidate to be the 9-1-1 opening gate. The Hus1-Rad1 interface was similar to PCNA in overall structure and hydrophobicity and opened up by an analogous rupture mechanism. However, the total binding energy ΔG_b_ of Hus1-Rad1 was higher than the energy of Rad9-Rad1. Furthermore, the affinities of Rad9-Rad1 and PCNA-PCNA were closely matched, despite pronounced dissimilarities in interface contacts and polar character. Thus, matching affinities may reflect the common energetic requirements of the clamp loading machinery to open PCNA and Rad9-Rad1.

Using external SMD forces as a surrogate of the clamp loader, we also identified the dominant pathways for interface disruption in PCNA and 9-1-1. In agreement with recent experimental work, we demonstrate the stochastic nature of clamp opening and observe an ensemble of opening pathways. We find that the overall directionality and cooperativity of the dominant pathways are distinct among the four clamp interfaces. Groups of contacts (especially charged and polar contacts) differentially stabilize the top and bottom regions of the Rad9-Rad1 and Rad9-Hus1 interfaces and determine the spatial and temporal patterns of breakdown. In Rad9-Rad1 and Rad9-Hus1, the dominant pathway involves unzipping of secondary structure elements in the ‘top-down’ direction. By contrast, the predominantly hydrophobic Hus1-Rad1 and PCNA interfaces are disrupted concertedly with no preferred directionality. Collectively, the results provide a framework for future experiments to examine the DNA clamp opening mechanisms. Specifically, single molecule micromanipulation experiments (with optical or magnetic tweezers) along with mutational substitution of residue pairs at the clamp interfaces could be used to probe the computationally identified pathways and establish the barriers and energy requirements for clamp opening.

## SUPPLEMENTARY DATA

Supplementary Data are available at NAR Online.

## FUNDING

National Science Foundation CAREER award [MCB-1149521 to I.I.] and Georgia State University start-up funds (to I.I.); Computational resources by a National Science Foundation XSEDE allocation (in part) [CHE110042] and an allocation (BIP007) at NERSC by the U.S. Department of Energy Office of Science [contract DE-AC02-05CH11231]. Funding for open access charge: National Science Foundation CAREER award [MCB-1149521 to I.I.].

*Conflict of interest statement*. None declared.

## Supplementary Material

Supplementary Data
