# Supplementary material for: Opening pathways of the DNA clamps proliferating cell nuclear antigen and Rad9-Rad1-Hus1
Source: Nucleic Acids Res. 2013 Sep 12;41(22):10020–31. doi: 10.1093/nar/gkt810 (PMC3905852; doi:10.1093/nar/gkt810)
Supplement: Supplementary Data [file supp_41_22_10020__index.html]

Opening pathways of the DNA clamps proliferating cell nuclear antigen and Rad9-Rad1-Hus1 — Opening pathways of the DNA clamps proliferating cell nuclear antigen and Rad9-Rad1-Hus1 — Supplementary Data 

# Opening pathways of the DNA clamps proliferating cell nuclear antigen and Rad9-Rad1-Hus1

## Supplementary Data

files

**Files in this Data Supplement:**

- Supplementary Data - pdf file
