# Supplementary material for: Opening pathways of the DNA clamps proliferating cell nuclear antigen and Rad9-Rad1-Hus1
Source: Nucleic Acids Res. 2013 Sep 12;41(22):10020–31. doi: 10.1093/nar/gkt810 (PMC3905852; doi:10.1093/nar/gkt810)
Supplement: Supplementary Data [file supp_gkt810_nar-01906-h-2013-File006.pdf]

Supplementary Information

Opening Pathways of the DNA Clamps Proliferating Cell Nuclear Antigen and Rad9-Rad1-Hus1

Xiaojun Xu[1], Carlo Guardiani[1†], Chunli Yan[1] and Ivaylo Ivanov[1*]

[1]Dept. of Chemistry, Georgia State University, Atlanta, Georgia 30302 USA

*Correspondence should be addressed to iivanov@gsu.edu

Contribution	PCNA/PCNA	3A1J		
	PCNA/PCNA	Rad9/Hus1	Hus1/Rad1	Rad9-Rad1
ΔE_{ele}	866.04 (46.68)	-411.36 (63.18)	-89.79 (35.88)	-694.78 (44.58)
ΔE_{vdw}	-80.65 (5.38)	-89.24 (8.55)	-84.54 (5.68)	-76.44 (5.51)
$\Delta G_{nonpolar}$	-11.68 (0.42)	-14.18 (0.96)	-11.46 (0.59)	-13.03 (0.56)
ΔG_{polar}	-827.27 (46.05)	437.34 (60.61)	109.80 (34.61)	724.07 (42.57)
$\Delta G_{sol}{}^{a}$	-838.95 (45.83)	423.16 (59.93)	98.34 (34.45)	711.04 (42.40)
$\Delta G_{ele}{}^{b}$	38.77 (10.49)	25.98 (13.44)	20.01 (7.49)	29.29 (11.87)
ΔG_b	**-53.56** (8.91)	**-77.44** (11.37)	**-75.99** (6.34)	**-60.18** (11.29)
ΔG_b Ratio	**1.00**	**1.45**	**1.42**	**1.12**
BSA (Å^2)	1555	1836	1475	1639

Table S1. Binding energy analysis (kcal mol^{-1}) for the DNA clamp interfaces in PCNA and 9-1-1 (3A1J model) [a]Polar/nonpolar (ΔG_{sol}=ΔG_{polar}+$\Delta G_{nonpolar}$) contributions to ΔG_b. [b]Electrostatic (ΔG_{ele} =ΔE_{ele}+ΔG_{polar}) contributions to ΔG_b. Calculation of ΔG_b does not explicitly consider entropy contributions. Standard deviations are shown in parentheses. Averaged buried surface areas (BSA) for the interfaces are given units of Å^2.

Contribution	3G65			
	PCNA/PCNA	Rad9/Hus1	Hus1/Rad1	Rad9-Rad1
ΔE_{ele}	866.04<br>(46.68)	-583.33<br>(49.33)	-97.19<br>(30.16)	-753.18<br>(54.20)
ΔE_{vdw}	-80.65<br>(5.38)	-91.96<br>(8.42)	-85.71<br>(4.85)	-80.15<br>(7.87)
$\Delta G_{nonpolar}$	-11.68<br>(0.42)	-15.48<br>(0.98)	-12.21<br>(0.59)	-12.76<br>(0.88)
ΔG_{polar}	-827.27<br>(46.05)	590.96<br>(46.84)	115.53<br>(29.59)	784.04<br>(54.66)
$\Delta G_{sol}{}^{a}$	-838.95<br>(45.83)	575.48<br>(46.56)	103.32<br>(29.32)	771.28<br>(54.10)
$\Delta G_{ele}{}^{b}$	38.77<br>(10.49)	7.62<br>(12.65)	18.33<br>(7.91)	30.86<br>(12.69)
ΔG_b	**-53.56**<br>(8.91)	**-99.82**<br>(10.87)	**-79.59**<br>(6.78)	**-62.05**<br>(11.58)
ΔG_b Ratio	**1.00**	**1.86**	**1.49**	**1.16**
BSA (Å2)	1555	2037	1556	1621

Table S2. Binding energy analysis (kcal mol^{-1}) for the DNA clamp interfaces in PCNA and 9-1-1 (3G65 model) ^{a}Polar/nonpolar (ΔG_{sol}=ΔG_{polar}+$\Delta G_{nonpolar}$) contributions to ΔG_b. ^{b}Electrostatic (ΔG_{ele} =ΔE_{ele}+ΔG_{polar}) contributions to ΔG_b. Calculation of ΔG_b does not explicitly consider entropy contributions. Standard deviations are shown in parentheses. Averaged buried surface areas (BSA) in units of Å^2.

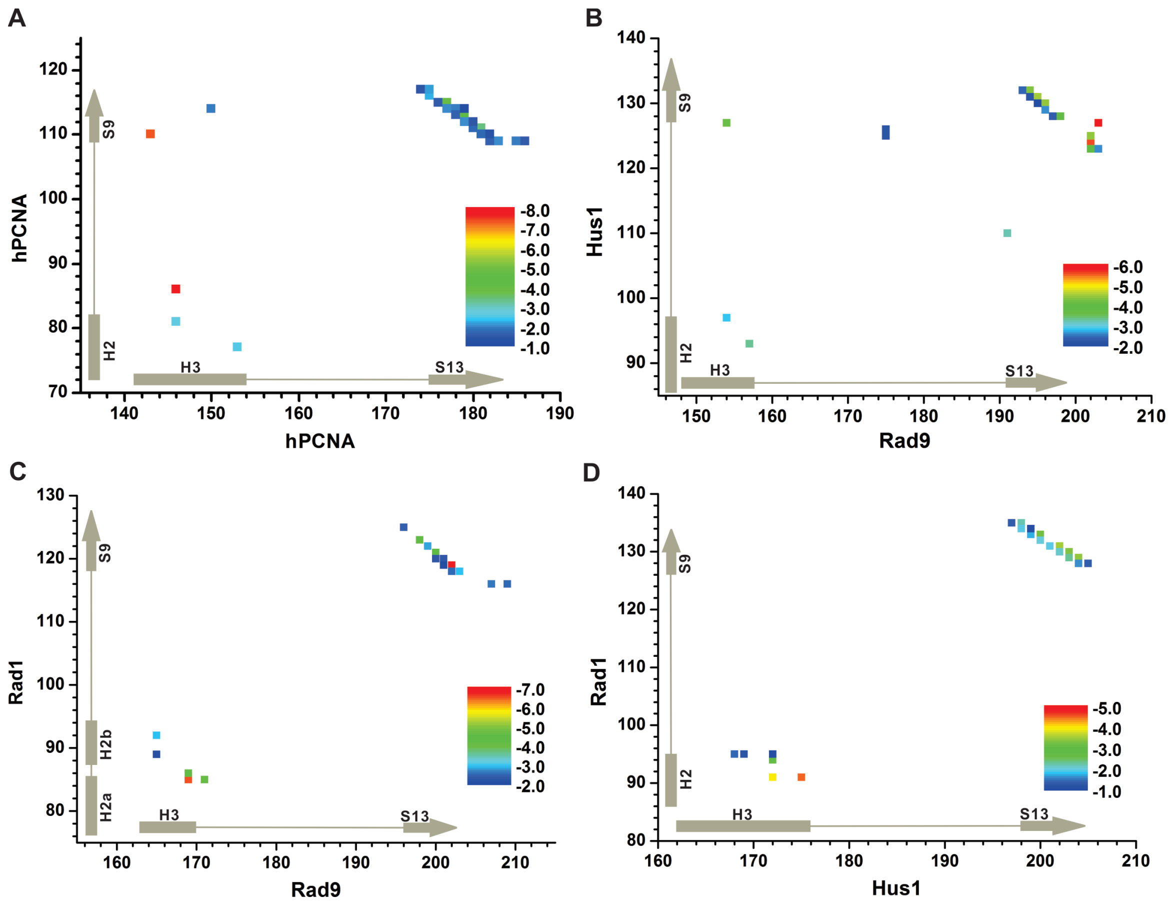

Figure S1. Significant (above-threshold) contacts from pairwise MM/GBSA binding energy decomposition Values for A) the PCNA interface; B) Rad9-Hus1 interface; C) the Rad9-Rad1 and D) the Hus1-Rad1 interface. Binding energies of residue pairs (in kcal/mol) are color-mapped on the panels from red to blue.
